# Evidence-Based Utility of Adjunct Antioxidant Supplementation for the Prevention and Treatment of Dermatologic Diseases: A Comprehensive Systematic Review

**DOI:** 10.3390/antiox12081503

**Published:** 2023-07-27

**Authors:** Jasmine Thuy Tran, Michael Joseph Diaz, Daphnee Rodriguez, Giona Kleinberg, Shaliz Aflatooni, Siri Palreddy, Parsa Abdi, Kamil Taneja, Sai Batchu, Mahtab Forouzandeh

**Affiliations:** 1 School of Medicine, Indiana University, Indianapolis, IN 46202, USA; 2 College of Medicine, University of Florida, Gainesville, FL 32610, USA; 3 College of Medicine, University of Central Florida, Orlando, FL 32827, USA; 4 College of Engineering, Northeastern University, Boston, MA 02115, USA; 5 Morsani College of Medicine, University of South Florida, Tampa, FL 33602, USA; 6 Department of Biology, Amherst College, Amherst, MA 01002, USA; 7 Faculty of Medicine, Memorial University, St. Johns, NL A1B 3V6, Canada; 8 Renaissance School of Medicine, Stony Brook University, Stony Brook, NY 11794, USA; kamil.taneja@stonybrookmedicine.edu; 9 Cooper Medical School, Rowan University, Camden, NJ 08103, USA; 10 Department of Dermatology, University of Florida, Gainesville, FL 32606, USA

**Keywords:** antioxidant supplementation, vitamins, reactive oxygen species, skin cancer, dermatology

## Abstract

Skin conditions are a significant cause of fatal and nonfatal disease burdens globally, ranging from mild irritations to debilitating diseases. Oxidative stress, which is an imbalance between reactive oxygen species and the cells’ ability to repair damage, is implicated in various skin diseases. Antioxidants have been studied for their potential benefits in dermatologic health, but the evidence is limited and conflicting. Herein, we conducted a systematic review of controlled trials, meta-analyses, and Cochrane review articles to evaluate the current evidence on the utility of antioxidant supplementation for adjunct prevention and treatment of skin disease and to provide a comprehensive assessment of their role in promoting dermatologic health. The Cochrane Library, PubMed, EMBASE, and Epistemonikos databases were queried. Eligibility criteria included (1) primary focus on nanoparticle utility for skin cancer; (2) includes measurable outcomes data with robust comparators; (3) includes a number of human subjects or cell-line types, where applicable; (4) English language; and (5) archived as full-text journal articles. A total of 55 articles met the eligibility criteria for the present review. Qualitative analysis revealed that topical and oral antioxidant supplementation has demonstrated preliminary efficacy in reducing sunburns, depigmentation, and photoaging. Dietary exogenous antioxidants (namely vitamins A, C, and E) have shown chemopreventive effects against skin cancer. Antioxidant supplementation has also shown efficacy in treating non-cancer dermatoses, including rosacea, psoriasis, atopic dermatitis, and acne vulgaris. While further studies are needed to validate these findings on a larger scale, antioxidant supplementation holds promise for improving skin health and preventing skin diseases.

## 1. Introduction

According to the Global Burden of Disease project, skin conditions are the fourth-leading source of nonfatal disease burden globally [1]. These conditions can range from mild irritations to chronic, debilitating diseases that significantly impact a person’s quality of life. Importantly, skin manifestations can give way to new diagnoses of otherwise occult systemic diseases, aiding in the timely initiation of important care and optimizing patient outcomes [2,3].

The management of dermatologic diseases requires a detailed evaluation of each patient and highly customized medical management plans with comorbidities and environmental factors in mind. The effects of oxidative stress on the skin and the potential utility of antioxidants in dermatology are being investigated in the more recent literature, highlighting a link between antioxidant imbalance and cutaneous diseases. In the skin, oxidative stress has been implicated in the pathogenesis of numerous skin diseases and disorders, including psoriasis, vitiligo, atopic dermatitis, pemphigus vulgaris, lichen planus, alopecia areata, melanoma, allergic contact dermatitis, acne vulgaris, solar elastosis, and pigmentary disorders. While the role of antioxidants is well-understood in diseases such as obesity, Alzheimer’s, and atherosclerosis, their use in numerous dermatologic diseases has not been well-established [4,5]

**Figure 1:**
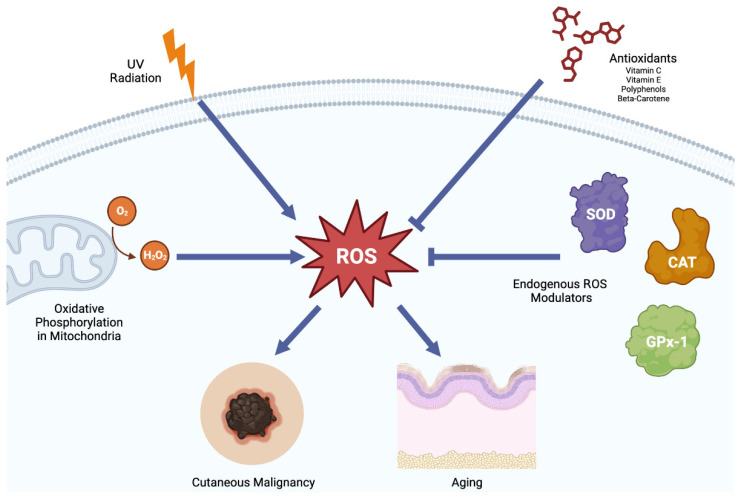
**Summary of the balance between reactive oxygen species and the endogenous antioxidant system.** ROS play a critical role in the development of skin cancer and dermatoses, such as skin aging and hyperpigmentation. ROS in the skin can build up with long-term exposure to UV radiation. They are also a natural by-product of a cell’s native metabolism. The cell has devoted many mechanisms to ensure that ROS levels are kept at a physiological state. However, when the system becomes overwhelmed, ROS can accumulate and lead the to the development of skin cancer and dermatoses. Antioxidants have shown to be an effective treatment in a wide range of skin issues. SOD: Superoxide dismutase. GPx-1 glutathione peroxidase-1. CAT: catalase. Figure created with BioRender.com.

Oxidative stress is a state of imbalance between the production of reactive oxygen species (ROS) and the ability of cells to detoxify or repair their damage. ROS are a natural byproduct of cellular metabolism and play a critical role in cellular signaling and homeostasis [6]. However, excessive ROS production can lead to oxidative stress, which can cause damage to cellular components, including lipids, proteins, and DNA, and ultimately lead to cellular dysfunction and death [7]. Antioxidants are compounds that can neutralize ROS and prevent oxidative damage (Figure 1).

Various antioxidants have been studied for their potential benefits to dermatologic health, including vitamins C and E, beta-carotene, astaxanthin, and selenium. Vitamin C is a water-soluble antioxidant that can scavenge free radicals and regenerate other antioxidants, such as vitamin E. Vitamin E is a fat-soluble antioxidant that can protect the skin’s lipid membranes from oxidative damage. Beta-carotene is a precursor of vitamin A, which is vital for skin health and immune function. Astaxanthin is a carotenoid with potent antioxidant and anti-inflammatory properties which has been shown to improve skin hydration, elasticity, and texture. Selenium is a trace element that plays a role in antioxidant defense and immune function and has been suggested to have a protective effect against skin cancer [8]. Although the potential benefits of antioxidant supplementation in dermatologic health are promising, the evidence is still limited and conflicting. Optimal dosages and formulations of antioxidants for dermatologic use are not commonly recognized. Moreover, excessive intake of certain antioxidants may have adverse effects and interfere with other physiological processes [9]. Thus, it is essential to carefully evaluate the efficacy and safety of antioxidant supplementation in specific dermatologic conditions and tailor the supplementation to individual needs, taking risk factors into consideration. 

It is also important to consider the efficacy of orally taken antioxidants, which involves considering several factors, including their ability to reach the skin in sufficient amounts and whether they may accumulate in other tissues instead. Studies indicate that orally administered antioxidants can reach detectable levels in the skin. For instance, Keen and Hassan demonstrated increased vitamin E levels in the skin following oral administration of vitamin E [10]. Similarly, Pullar et al. showed increased vitamin C levels in the skin after oral supplementation with vitamin C [11]. Certain antioxidants, such as vitamin C and vitamin E, have been found to accumulate in the skin layers requiring antioxidation. However, the distribution and accumulation of orally taken antioxidants in the skin may vary depending on the antioxidant and individual factors. Additionally, some antioxidants may be distributed and accumulate in other tissues, as shown in studies by Darvin et al. with beta-carotene, which increased levels in the skin, plasma, and adipose tissue [12].

In this systematic review, we aim to evaluate the current evidence regarding the utility of antioxidant supplementation for the prevention and adjunct treatment of various skin diseases and disorders. We will review the pathophysiology of oxidative stress in dermatologic conditions and summarize the findings from preclinical and clinical studies on the effects of antioxidant supplementation on skin health and function. We will also discuss the potential mechanisms of action of different antioxidants, the safety and tolerability of supplementation, and the implications for clinical practice and future research. Overall, this review aims to provide a comprehensive and evidence-based evaluation of the role of antioxidant supplementation in promoting dermatologic health.

## 2. Methods

### 2.1. Study Design

A systematic review of controlled trials, randomized controlled trials (RCTs), meta-analyses, case reports, and review articles was conducted in accordance with the latest (2020) Preferred Reporting Items for Systematic Reviews and Meta-Analyses (PRIMSA) guidelines [13]. This review protocol was registered in the international prospective register of systematic review (PROSPERO) (CRD42023416336).

### 2.2. Search Strategy

The Cochrane Library, PubMed, EMBASE, and Epistemonikos databases were broadly queried on 10 March 2023 to retrieve all relevant articles since the database’s inception. The main keyword search terms were “antioxidant” AND “skin disease” OR “skin cancer”. Queries were restricted to the Title and Abstract fields (“[tiab]”). Search records were maintained with Covidence (www.covidence.org, accessed on 10 March 2023), a web-based collaborative platform designed to streamline and automate many aspects of the systematic review process [14].

### 2.3. Eligibility Criteria

All initial search results were subjected to the following inclusion criteria: (1) has a primary focus on antioxidant supplementation for dermatologic conditions and/or diseases, (2) includes measurable outcomes data with robust comparators (i.e., changes compared with baseline or comparison to age-matched, healthy human patients or non-exposed controls), (3) includes a number of human subjects or cell-line types, where applicable. Criteria for exclusion were (1) non-English articles, (2) Abstract-only text, and (3) not-yet-published clinical trials.

### 2.4. Data Extraction

**Figure 2:**
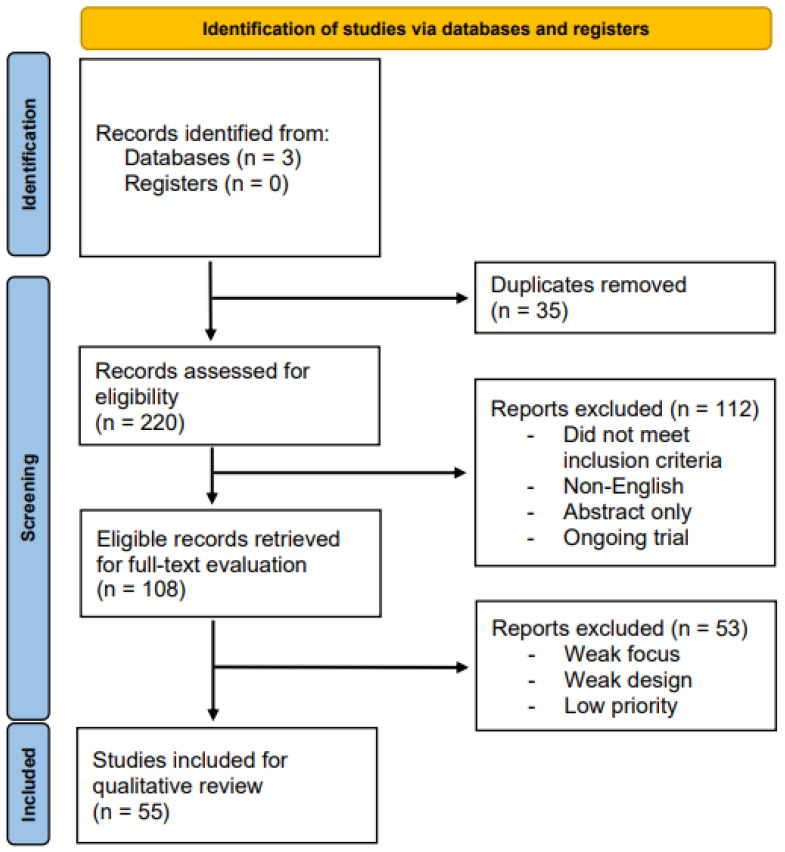
PRISMA flow diagram.

The initial sensitivity search returned 255 total records. Following the elimination of duplicates, 220 articles underwent independent Abstract and Title screening for eligibility assessment by three of the authors (BG, DG, and GK). From these records, 108 articles were deemed eligible for full-text review. After a thorough examination of the relevant studies, 55 studies were deemed suitable for qualitative synthesis. Any discrepancies in the selection process were resolved through discussion or by seeking the input of an impartial third party (JTT). Figure 2 depicts the PRISMA selection process flowchart used for this systematic review.

## 3. Results

### 3.1. Primer on Antioxidant Supplementation

Exposure of the skin to ultraviolet radiation (UVR) generates reactive oxygen species (ROS) that can deplete endogenous antioxidants and cause acute and chronic changes to the skin, such as sunburns, depigmentation, photoaging, and cutaneous malignancy [15,16].

Several studies have shown the clinical efficacy of topical and oral antioxidant supplementation on mitigating UV-mediated damage by neutralizing free radicals and protecting the skin. Vitamins C and E are naturally strong antioxidants and can be combined with other antioxidant supplementation to promote cutaneous radical scavenging activity after UV radiation, as shown in many studies [16,17]. One such study used electron paramagnetic resonance in subjects who consumed a vitamin C supplement or a chokeberry peel extract (Aronia) [17]. Oral supplementation with vitamin C and Aronia significantly increased the radical scavenging capacity of the skin by 22% and 23%, respectively [17]. Another study demonstrated that a stable and permeable vitamin C derivative, tetra-isopalmitoyl ascorbic acid (VC-IP), could effectively suppress UVB-induced skin pigmentation, reduce melanocyte proliferation, and reduce oxidative stress [18]. Additionally, a study analyzed the effect of long-term oral intake of vitamin D and E analogues for 3 months and found a significant reduction in sunburn reaction and significantly fewer thymine dimers following UVB exposure [16]. Further, the effects of a topical antioxidant mixture consisting of vitamin C, ferulic acid, and phloretin, a natural phenol, showed the increases in sunburn cell formation and thymine dimer formation to be attenuated in subjects treated with the topical antioxidant cream compared with the vehicle control [19]. Pretreatment of the skin with the cream also blocked the suppression of CD1a-expressing Langerhans cells after UV irradiation [19]. The unique topical antioxidant mixture of vitamin C and ferulic acid was further analyzed but with vitamin E instead of phloretin [20]. Subjects who received a topical antioxidant solution containing 15% vitamin C, 1% vitamin E, and 0.5% ferulic acid (CEFer) experienced a significantly decreased erythema and total number of sunburn cells. Immunohistochemical analyses also revealed a significant decrease in thymine dimer formation [20]. 

Tocopherols and tocotrienols, two major forms of vitamin E, were also studied [21]. The prophylactic efficacy of a topical agent containing tocopherols 10% and tocotrienols 0.3% was evaluated [21]. Erythema was significantly lower in areas treated with the topical vitamin E formulation compared with those treated with the simple vehicle or vitamin A [21]. Thus, high concentrations of tocotrienols and tocopherols in a topical formulation show a promising strategy to reduce photoinduced skin damage. Vitamin E is also a popular active ingredient found in sunscreens in the form of alpha-tocopherol acetate [22]. A study evaluated if topical application of alpha-tocopherol acetate is absorbed in human skin and metabolizes to alpha-tocopherol, the free form of vitamin E, since it is known that the free unesterified form of alpha-tocopherol significantly reduces experimental UVB carcinogenesis [22]. The study found no evidence of the systemic availability or biotransformation of topically applied alpha-tocopherol acetate to the free form of alpha-tocopherol in plasma or skin, which may be concerning since many commercial sunscreens and lotions contain this synthetic form of vitamin E, which does not protect against UV radiation [22].

Additional antioxidant supplements have been investigated for their photoprotective capacities. Polyphenols, such as the natural botanical compound resveratrate, have shown powerful antioxidant capacities [23]. Resveratrate-treated skin decreased the appearance of erythema and resulted in a significant reduction in the formation of sunburn cells after UVR exposure [23]. Further, topical 12.5% melatonin cream also demonstrated the ability to protect against erythema induced by UVR from natural sunlight by acting as a radical scavenger [24]. Additionally, reduced glutathione (GSH) has important properties in the protection of UVB-induced damage to DNA [25]. A study evaluated the efficacy of the application of a cream containing GSH conjugated to a long-chain polyunsaturated fatty acid called S-linolenoyl-glutathione (Lin-GSH) and found that Lin-GSH was able to significantly inhibit erythema [25]. A portion of this observed protective effect exerted by the Lin-GSH cream was attributed to molecular absorption of the UV radiation by multiple double bonds of the hydrocarbon chain of unsaturated fatty acids [25]. Additionally, topical administration of a dried melon juice concentrate, particularly rich in superoxide dismutase (SOD), showed promising results in reducing UV-induced cytotoxicity [15]. The topical melon juice concentrate demonstrated a significant increase in minimal erythema dose (MED), defined as the amount of UV radiation that will produce noticeable redness on an individual’s skin 24 h after sun exposure [15]. Topical melon concentrate application significantly decreased the formation of sunburn cells, or keratinocytes, undergoing apoptosis after irradiation in human skin explants compared with the placebo cream [15]. The level of glutathione peroxidase-1 (GPx-1), catalase (CAT), and SOD were significantly increased in irradiated skin explants after topical application of the active cream when compared with untreated explants and placebo-irradiated skin [15]. Further, the combination of antioxidants with DNA-repair enzyme complexes has shown improvement in the genomic and proteomic integrity of skin cells exposed to UVR [26].

Three studies analyzed the efficacy of oral supplementation with beta-carotene, a known antioxidant, compared with sunscreen for protection against skin cancer [27,28,29]. Daily beta-carotene supplementation did not reduce the incidence of basal cell carcinoma (BCC) and squamous cell carcinoma (SCC) after a 4.5-year follow-up compared to that with daily sunscreen with a sun protection factor of 15 [27]. A separate long-term placebo-controlled trial found that 12 years of beta-carotene supplementation did not affect the incidence of BCC and SCC [28]. Further, a separate study conducted over four years randomly assigned subjects to daily sunscreen use or beta-carotene supplement, and the incidence of solar keratoses (SKs) was measured since SK is a strong determinant of skin cancer. It was found that sunscreen was able to slow the rate of SK acquisition, but beta-carotene supplementation did not influence the occurrence of SKs [29].

Landmark articles describing the general utility of antioxidant supplementation for the treatment of dermatologic disease, to date, are summarized in Table 1.

### 3.2. Non-Cancer Utility 

**Figure 3:**
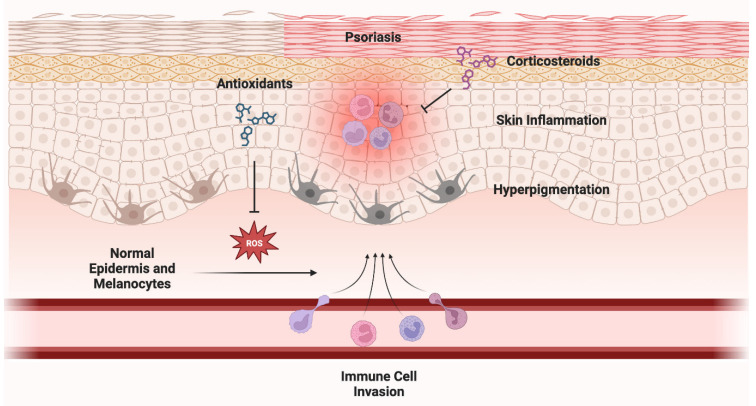
**Role of ROS in the development of non-cancer skin conditions.** ROS can lead to 2 major outcomes: (1) skin inflammation and (2) hyperpigmentation through activation of melanocytes. ROS have been implicated in the development of erythema, psoriasis, rosacea, and acne vulgaris. Studies have shown that antioxidants (sometimes combined with corticosteroids) can drastically improve these conditions. Figure created with BioRender.com.

Numerous studies have demonstrated the efficacy of antioxidant supplementation against various dermatoses [30,31,32]. For rosacea, oral diammonium glycyrrhizinate combined with oral clarithromycin, or isotretinoin, was more effective and rapid than clarithromycin or isotretinoin alone in treating rosacea primarily characterized by papules and pustules [30]. Turmeric has demonstrated antioxidant and anti-inflammatory effects and increases skin moisture. It has proven effective in reducing thickness, erythema, pruritus, burning, and pain in psoriasis lesions and improving radiodermatitis lesions [31]. Moreover, a trial led by Dall’oglio and colleagues reported preliminary evidence that prescription-strength (15%) azelaic acid with 1% dihydroavenanthramide D yielded a significant decrease in inflammatory lesions and erythema associated with inflammatory rosacea, which could serve as an adjunct to the current therapeutic interventions for managing inflammatory rosacea [32]. See Figure 3 for exemplification of the role of ROS in the development of non-cancer skin conditions. Landmark articles describing the utility of antioxidant supplementation for the treatment of non-cancer dermatologic conditions are summarized in Table 2.

Antioxidant supplementation has also evidenced efficacy for psoriasis. Studies suggest oral CoQ10 adjuvant therapy may be an effective supplement as it improves the correlation between the Psoriasis Area and Severity Index (PASI) and the Dermatology Life Quality Index (DLQI) after 12 weeks of treatment [33]. A second indicated antioxidant is astilbin, a component of Rhizoma smilacis glabrae that may function against autoimmune diseases [34]. Topical administration of low-dose astilbin has reduced psoriasis-like lesions and psoriasis-specific cytokine expression, thus attenuating psoriasis [34]. Oral supplementation with turmeric tonic was also shown to significantly reduce erythema, scaling, and induration of lesions and improve the overall quality of life in patients [35]. Compared with the control group, turmeric tonic resulted in significant improvements in the management of scalp psoriasis [35]. A review on vitamin D effectiveness in patients with chronic plaque psoriasis found that when vitamin D analogues were used on the body, they were significantly more effective than the placebo [36]. For scalp psoriasis, calcipotriol monotherapy alone was considerably less effective than corticosteroids; additionally, potent corticosteroids were less likely than calcipotriol to cause local adverse reactions such as burning or irritation [36]. However, for both body and scalp psoriasis, the combined treatment of topical corticosteroids with vitamin D analogues performed significantly better than vitamin D analogues or corticosteroids alone, with minimal side effects [36]. Unlike vitamin D, oral vitamin C has demonstrated insignificant effects against psoriasis [37]. Although oral vitamin C supplementation directly correlated with GSH levels, a weak, negative, and negligible correlation was found between vitamin C and PASI scores [37]. 

Additional supplements have been investigated for various dermatoses. The herbal supplements Majoon Ushba (MU) and Marhaam Raal (MR), which have antioxidant, antimicrobial, antifungal, and anti-inflammatory properties, were utilized topically against tinea corporis [38]. MU and MR demonstrated equal effectiveness against tinea corporis compared to traditional/conventional treatments as measured by erythema, scaling, margins, size of the lesion, and itching [38]. The herbal cream containing silymarin and the antioxidant fumaria officinalis may help improve the severity and symptoms of atopic dermatitis in patients, comparable to the control mometasone cream [39]. Additionally, vitamins D(3) and E (600 IU synthetic all-rac-ɑ-tocopherol) may be beneficial against atopic dermatitis [40]. Javanbakht et al. showed significant reductions in SCORing Atopic Dermatitis (SCORAD) in groups receiving vitamin D, E, or D and E [40]. Further, the antioxidant 4% gumamela leaf extract ointment may be effective in venous leg ulcer (VLU) closures [41]. Findings showed that the gumamela leaf extract ointment used with compression stockings closed VLUs in less than 12 weeks in 83% of patients (N = 12) [41]. Oral curcumin supplementation was also investigated against chronic pruritis induced by sulfur mustard (SM) exposure [42]. Curcumin supplementation was associated with significant reductions in pruritus severity [42]. Polyphenon E, a green tea catechin, has also been indicated as a treatment against HPV warts. Polyphenon E 15% and 10% ointment demonstrated significant effectiveness in the complete clearance of baseline and new external anogenital warts (EGWs), as well as possibly lowering recurrence rates [42]. Polyphenon, however, was not compared with the principal treatment, imiquimod [42].

Moreover, several studies examined the effects of antioxidant supplementation against acne vulgaris. Multiple vitamin C analogues were investigated since vitamin C is too unstable [43,44,45]. A study has indicated Sodium L-ascorbyl-2-phosphate (APS) as effective as a monotherapy for the treatment of acne, as it showed improvement in 61% of subjects as measured by the investigator (n = 50) [43]. Further, vitamin C capsules taken with doxycycline are significantly more efficient than doxycycline alone therapeutically and in reducing serum levels of IL-8, IL-1β, IFN-γ, TNF-α, and TLR-2 [44]. Sodium ascorbyl phosphate (SAP) at 1% demonstrated a strong antimicrobial effect as well as effectiveness in the treatment and prevention of acne vulgaris with minimal side effects [45]. Lastly, topical vitamin E proved ineffective in reducing the side effects of isotretinoin for the treatment of acne vulgaris [46]. 

### 3.3. Anti-Cancer Utility

**Figure 4:**
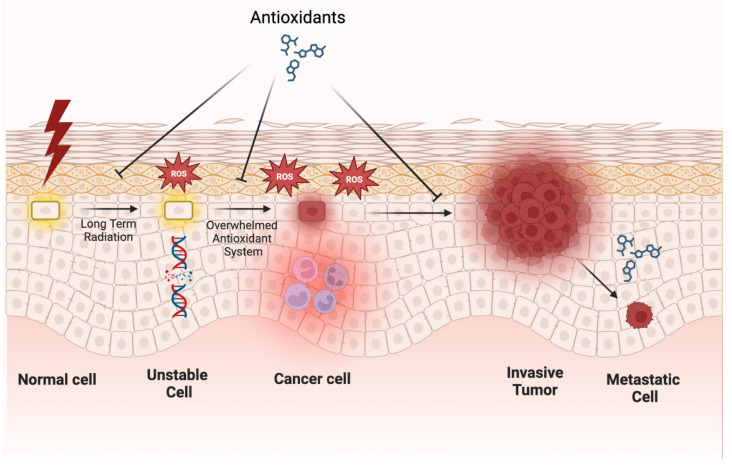
**Role of ROS in the development of skin cancer.** Long-term UV radiation can lead to ROS, which can lead to DNA damage. The combination of ROS and DNA damage can overwhelm a cell’s inherent antioxidant system, eventually leading to a cancer cell. Antioxidants have been shown to greatly inhibit the development of skin cancer. Interestingly, oxidative stress can inhibit the development of metastatic cells, and antioxidants can promote the development of these cells. Figure created with BioRender.com.

Skin cancer can arise when skin cells are exposed to highly unstable reactive oxygen species (ROS), which, in excess, are associated with the proliferation of cancer cells. ROS can be generated after the skin is exposed to ultraviolet radiation (UVR), leading to oxidative stress if antioxidant levels are too low to compensate for increased DNA damage caused by ROS. While the antioxidant system in the skin is normally well-equipped to manage oxidative stress, chronic or excessive exposure to UVR or other oxidizing agents can overwhelm the system and result in melanoma [47]. See Figure 4 for a representation of the role of ROS in the development of skin cancer.

The antioxidant system acts as a defense against melanoma in that it will either work to prevent the generation of ROS and eliminate any free radicals, which are similarly highly reactive, or it will function through a repair circuit to remove damaged biomolecules, preventing their accumulation and the subsequent effect on cell function [47]. A key component of the antioxidant defense system is manganese-superoxide dismutase (MnSOD). Poswig et al. found that the adaptive antioxidant response of manganese-superoxide dismutase can be induced following repeated exposure to UVR, with increasing induction of the MnSOD antioxidant mRNA quantity and degree of activity observed after UVR in fibroblasts [48]. While the MnSOD antioxidant seems to be linked positively to UVR, not all individuals’ MnSOD increases with UVR. Furthermore, Poswig et al.’s association between MnSOD levels and UVR does not necessarily imply all antioxidants respond identically to UVR exposure. Using mouse skin, Fuchs et al. showed that acute UVR exposure led to a decrease in glutathione reductase and catalase activity in mouse skin, but to insignificant changes in superoxide dismutase and glutathione peroxidase, all of which are antioxidants [49]. 

In addition to endogenous antioxidants, dietary exogenous antioxidants such as vitamins A, C, and E may offer a source of protection against melanoma, for which UVR exposure is a major risk factor. Elmore (2005) reported evidence that magnesium ascorbyl phosphate (which contains the exogenous antioxidant ascorbic acid) administered immediately after exposure in hairless mice significantly delayed skin-tumor formation and hyperplasia induced by chronic exposure to UVR and that ascorbic acid applied to mice and pig skin prior to UVR reduced skin-cancer-related damage [50]. Evidence of magnesium ascorbyl phosphate’s toxicity was obtained at ingested levels significantly higher than what one would encounter in typical cosmetic products with magnesium ascorbyl phosphate or ascorbic acid.

Another exogenous antioxidant, silymarin, has been shown to have chemopreventive effects against chemical carcinogenesis as well as photocarcinogenesis in various animal tumor models [51]. Topical treatment of silymarin inhibited several tumor promoters and showed evidence of preventing UVR-induced skin carcinogenesis [51]. Important to note is the difference in the antioxidant’s administration route within these studies. While silymarin was applied topically, magnesium ascorbyl phosphate was injected peritoneally in animal models. The means of delivery may have some effect on the antioxidant response. Another limitation to these studies is that most were conducted on animal or human in vitro models. Human in vivo studies are preferred for studying the utility of antioxidant supplementation in humans. In human subjects, dietary beta-carotene has been found to manage UVR-induced DNA damage in the skin in vivo [52]. Other dietary antioxidants such as curcumin and nobiletin, found naturally in turmeric and citrus fruits, respectively, were shown to have an anti-tumor initiating effect when used to treat tumors experimentally initiated by the reactive oxidant peroxynitrite [53]. In animal models, topical treatment with or oral consumption of green tea polyphenols (GTP) inhibited chemical carcinogen- or UVR-induced skin carcinogenesis [54]. High concentrations of selenium applied topically through serums were associated with a lower risk of basal or squamous cell carcinoma [55].

While endogenous and dietary antioxidants have been shown to protect against cancer, some more recent contradictory clinical findings have found a carcinogenic effect of antioxidants. Le Gal et al. found oral administration of the antioxidant N-acetylcysteine (NAC) increased lymph node metastases in an endogenous mouse model of malignant melanoma [56]. Additionally, NAC and the soluble vitamin E analogue Trolox markedly increased the migration and invasive properties of human malignant melanoma cells, but notably did not affect their proliferation [56]. Another study found that a combination of dietary antioxidants [120 mg vitamin C (sodium ascorbate), 30 mg vitamin E (dl-α-tocopherol), 6 mg β-carotene, 100 μg selenium (selenium-enriched yeast), and 20 mg zinc (zinc gluconate)] increased the risk of melanoma in female subjects, but interestingly not in male subjects, possibly attributed to metabolism differences between men and women [57]. Further linking dietary antioxidants and the risk of cancer are findings that the supplementation of vitamins C and A accelerates malignant melanoma metastasis in mice [58]. Piskounova et al. provide additional evidence that antioxidants can promote cancer metastasis. In mouse models of melanoma, the authors found that levels of oxidative stress were higher in circulating cancer cells than in primary-tumor cancer cells [59]. They found oxidative stress interfered with the formation of metastatic tumors, contrary to the current line of thinking that oxidative stress is solely carcinogenic. Treating these mice with antioxidants decreased oxidative stress in the circulating cancer cells and increased their ability to metastasize. In another study investigating the potential delayed effect of oral antioxidant supplementation on skin cancer incidence, after a 5-year post-intervention follow-up, it was found that the elevated melanoma risk associated with the original antioxidant treatment group receded when antioxidant supplementation was stopped, suggesting a potential causative role of antioxidant supplementation in the appearance of melanomas [60]. Many of these studies find a link between antioxidants and melanoma risk, but more research is needed to understand the different roles antioxidants have in various contexts, as well as to broaden the investigation of the effect of antioxidants to UVR-induced skin cancers other than melanoma.

Landmark articles describing the utility of antioxidant supplementation for the treatment of skin cancer are summarized in Table 3.

### 3.4. Emerging Research

There is a magnitude of new advances in antioxidant supplementation. Burke and colleagues found that concentrated ascorbic acid in DMSO is a well-tolerated, inexpensive, and easy-to-use topical preparation that was superior to 5% topical imiquimod at 8 weeks and non-inferior at 12 weeks for low-risk nodular and superficial BCC [61,62,63]. The presence or absence of residual tumor was determined using a 2 mm punch biopsy after 8 weeks of treatment. Non-resolved lesions that received topical imiquimod treatment were treated an additional 4 weeks. Ascorbic acid was also associated with fewer adverse effects than imiquimod, although there was no significant difference in recurrence during a 30-month observation period [61]. This new topical treatment could potentially reduce costs and improve outcomes for BCC, particularly for lesions on the face where surgical scarring and cosmesis are significant concerns. While the study did not explore the mechanism of action of ascorbic acid on nodular BCC, which can extend several millimeters in depth, Burke and Bailie hypothesized that indirect inflammation-related effects may be involved [61]. 

Larger controlled clinical trials are needed to confirm these preliminary findings [62,63,64], with desired aims to identify cotreatment options and ideal dosing regimens informed by a better understanding of the mechanisms of action [64,65,66]. Furthermore, Farajzadeh et al. recently evaluated the effectiveness of carboxytherapy combined with narrowband-ultraviolet B (NB-UVB) in treating vitiligo, with results indicating that combination therapy resulted in significantly higher rates of repigmentation compared with NB-UVB alone, and there were no significant differences based on the demographic or clinical features of the patients [67].

Several double-blind clinical trials showcase higher clinical efficacy through probiotic supplementation [68,69]. The objective of one study was to assess the efficacy of a food supplement containing specific strains of probiotics in ameliorating atopic dermatitis (AD) symptoms and skin conditions in adult participants [69]. The research involved a randomized controlled trial with 80 adults exhibiting mild-to-severe AD symptoms who received either a placebo or the probiotic mixture for a period of 56 days. The group who received the probiotics experienced an improvement in skin smoothness, moisturization, and self-perception, as well as a reduction in inflammatory markers linked to AD. This positive trend continued for a month after the cessation of the supplement. The study concluded that the administration of the chosen probiotic strains led to a prompt and sustained improvement in AD-related symptoms and skin conditions. A second study by Moludi et al. aimed to evaluate the effects of probiotics on the quality of life, oxidative stress, inflammatory markers, and clinical outcome of psoriasis patients [68]. Fifty patients were randomized into two groups, one receiving a probiotic drink with Lactobacillus strains for eight weeks and the other receiving a placebo. The probiotic group showed significant improvements in depression scores, quality of life, psoriasis area and severity index, psoriasis symptom scale, total antioxidant capacity levels, and decreased levels of inflammatory markers. The study suggests that probiotics may improve the quality of life and inflammatory biomarkers in psoriatic patients. Further studies are needed to establish probiotics as a routinely prescribed therapy for inflammatory dermatoses. The range of probiotic research seeking to improve dermatological diseases can benefit from future research in a few key areas. The development of specific probiotic formulations may lead to higher success. The creation of such formulations is dependent on an understanding of the probiotic’s mechanism of action as well as the role of the gut microbiome in mediating probiotic effects. By better characterizing how probiotics work and what influences their effect, further research can personalize and specialize probiotic therapy, further leading to improved clinical outcomes.

Landmark articles describing emerging findings in antioxidant supplementation are summarized in Table 4.

## 4. Discussion

The role of antioxidants in the prevention and treatment of numerous dermatoses has been examined by recent papers, highlighting a link between antioxidant imbalance and dermatological diseases. The skin serves an essential role as the body’s first line of defense against pathogens, toxins, and ultraviolet (UV) radiation [8]. Its frequent exposure to environmental stressors, including UV radiation, pollution, and lifestyle habits such as smoking, can lead to oxidative stress and damage to the skin that increases the risk of cutaneous diseases [70]. Oxidative stress occurs when an imbalance exists between the production of ROS and antioxidant defense mechanisms, where ROS levels exceed the body’s antioxidant defenses, causing cellular damage [71]. This damage can lead to the onset and/or progression of dermatological diseases and disorders, such as photoaging, pigmentation disorders, atopic dermatitis, psoriasis, and skin cancer [72]. Recent studies depict the promising utility of antioxidant supplementation in both the prevention and treatment of skin diseases and disorders through the promotion of maintaining the physiological balance of the skin barrier by reducing oxidative stress and inflammatory processes [73]. 

Several antioxidant agents, such as vitamin C, vitamin E, polyphenols, and carotenoids, have demonstrated efficacy in neutralizing ROS to prevent oxidative damage associated with inducing or irritating various dermatoses [8,74]. Previous research has identified a role for oxidative stress in pigmentation disorders, such as melasma and post-inflammatory hyperpigmentation, where ROS activate melanocytes, leading to increased melanin production and subsequent hyperpigmentation [75]. This led to investigations of numerous antioxidants for melasma therapy that suggested the use of antioxidants as a monotherapy or in combination with other melasma therapies [76]. Inflammatory skin diseases, such as atopic dermatitis and psoriasis, have found that oxidative stress is involved in the pathogenesis of these diseases [77,78]. 

Multiple studies have investigated the use of antioxidant supplements in the prevention of skin cancer. Antioxidants neutralize free radicals and enhance DNA enzyme repair systems [9]. The DNA-repair capacity of human skin cells is, therefore, related to the carcinogenesis initiation probability. Accumulating evidence depicts how dietary changes and supplementation with specific micronutrients may help prevent oxidative stress and the formation of free radicals that promote the skin-damage process [47]. A randomized controlled trial of 386 women found that oral supplementation with selenium, vitamin E, and beta-carotene reduced the incidence of basal cell carcinoma [79]. Since the human body cannot synthesize exogenous antioxidants like vitamins C and E, they must be obtained through the diet [80]. Although endogenous and dietary antioxidants have demonstrated some efficacy in protecting against cancer, some recent clinical studies reported contradictory findings, with antioxidants having a carcinogenic effect. These studies note how chronic antioxidant consumption could foster harmful side effects that increase the risk of developing malignancies [81]. Moreover, emerging research in antioxidant supplementation characterizes promising new therapeutics in the form of topical treatments, combination therapy, and probiotic supplementation that offer antioxidant properties aimed at improving outcomes of dermatological diseases [61,68,69].

A primary limitation of this present review is the lack of available clinical data conducted on a larger scale to strengthen conclusions drawn about the role of antioxidant supplementations. It is important to note that many of these studies revealed an in vitro role of antioxidants and investigated biomarkers in different tissues employing different methods. The results of in vitro study may not necessarily correspond to clinical outcomes [8]. Additionally, although some of the studies, especially the ones on topical antioxidants, are well-designed, they may contain biases as they are promoted by cosmetic enterprises, which can influence the research agenda in favor of corporate interest. Additional long-term studies are warranted to validate our findings and shed light on the potential clinical and therapeutic implications. With these limitations in mind, some considerations on the role of antioxidant supplementation in various dermatoses can be postulated from the literature data available. Antioxidant supplementation is a promising approach for improving skin health and preventing the development of skin diseases and disorders.

## 5. Conclusions

Here, we provided a systematic appraisal and critique of the available body of relevant literature on antioxidant supplementation for dermatologic health. This review collates varying levels of evidence on the effects of antioxidants in the prevention and treatment of dermatological diseases and disorders, revealing notable associations between the imbalance of antioxidant systems and various dermatoses. Existing data are accumulating on the efficacy of antioxidant supplementation in promoting skin health. Strengths of this review include the adherence to current PRISMA guidelines and the inclusion of multiple literature types. Potential limitations include the lack of a formal qualitative analysis and data synthesis. Further long-term studies on a larger scale are needed to corroborate the large wealth of literature data available on the role of antioxidants in dermatology to determine the implications for clinical applications, and we hope that future research will expand on these salient aspects.

## Figures and Tables

**Table 1:** Summary of studies retrieved to develop a primer on the utility of antioxidant supplementation for dermatologic diseases (n = 15).

Author, Year	Study Design	Key Findings
Egoumenides, 2018 [15]	Randomized controlled trial	- Melon concentrate application and/or supplementation increased MED (*p* < 0.0001) and the endogenous antioxidant enzymes (*p* < 0.01) - Melon concentrate reduced total number of sunburn cells (*p* < 0.01)
Placzek, 2005 [16]	Controlled trial	- Oral supplementation with vitamins C and E for 3 months significantly reduced UVB-irradiated skin damage, as detected by significantly fewer thymine dimers after UVB exposure (*p* < 0.05)
Meinke, 2012 [17]	Randomized controlled trial	- Oral supplementation with vitamin C and Aronia, a chokeberry peel extract, significantly increased the antioxidant activity of the skin by 22% and 23%, respectively (*p* < 0.05)
Ochiai, 2006 [18]	Controlled trial	- Tetra-isopalmitoyl ascorbic acid (VC-IP) reduced the production of interleukin-1alpha and prostaglandin E2 and suppressed the elevation of intracellular peroxide in UVB-irradiated keratinocytes
Oresajo, 2008 [19]	Randomized controlled trial	- Topical antioxidant mixture containing vitamin C, ferulic acid, and phloretin significantly reduced total sunburn cells, thymine dimer formation, matrix metalloproteinase-9 expression, and p53 protein expression (*p* < 0.01) - Pretreatment with the antioxidant composition blocked the suppression of CD1a-expressing Langerhans cells (*p* < 0.01) - Pretreatment with the antioxidant composition significantly reduced erythema after UV exposure (*p* < 0.01)
Murray, 2008 [20]	Controlled trial	- Topical antioxidant solution containing vitamins C and E and ferulic acid significantly decreased erythema, total number of sunburn cells, thymine dimer formation, and p53 expression (*p* < 0.01)
Pedrelli, 2012 [21]	Controlled trial	- Statistically significant difference between erythema and itch reactions of areas treated with vitamin E and those treated with a simple vehicle after UVB radiation (*p* < 0.05)
Alberts, 1996 [22]	Controlled trial	- Topically applied alpha-tocopherol acetate was substantially absorbed in the skin, but there was no evidence of conversion within skin to free alpha-tocopherol
Wu, 2013 [23]	Randomized controlled trial	- 1% resveratrate alone and 1% resveratrate in combination with a preparation of antioxidants significantly reduced the total number of sunburn cells (*p* < 0.05)
Scheuer, 2017 [24]	Randomized controlled trial	- Topical melatonin cream significantly decreased erythema reaction 8 h after exposure to natural sunlight
Grandi, 2019 [25]	Randomized controlled trial	- Topical application of Lin-GSH 2% cream significantly increased MED compared with placebo
Emanuele, 2014 [26]	Controlled trial	- TPF50 consisting of SPF 50 sunscreen, a liposome-encapsulated DNA repair complex, and a potent antioxidant complex reduced cyclobutene pyrimidine dimers and protein carbonylation
Green, 2000 [27]	Randomized controlled trial	- There was no statistically significant difference in basal cell carcinoma rates in participants who used sunscreen daily and participants who did not use sunscreen daily - There was no statistically significant difference in rates of basal cell carcinoma and squamous cell carcinoma in participants who received beta-carotene supplements and those who received placebo
Frieling, 2000 [28]	Randomized controlled trial	- 12 years of supplementation with 50 mg of beta-carotene had no effect on the incidence of a first nonmelanoma skin cancer, BCC, or SCC
Darlington, 2003 [29]	Randomized controlled trial	- The ratio of solar keratoses counts in 1994 relative to 1992 was lower in adults randomized to daily sunscreen use compared with adults randomized to discretionary sunscreen use - No effect of the rate of change of prevalent solar keratoses counts was seen in adults taking 30 mg of beta-carotene versus those taking placebo tablets

**Table 2:** Summary of studies retrieved to remark on the utility of antioxidant supplementation for non-cancer skin conditions (n = 17).

Author, Year	Study Design	Key Findings
Xie, 2022 [30]	Randomized controlled trial	- Groups given oral diammonium glycyrrhizinate (DG) concurrently with oral clarithromycin and isotretinoin indicated more effective symptom relief in patients with rosacea compared with groups not receiving DG. Results indicated DG was effective in reducing symptom scores quicker and more effectively. - No significant difference between the groups with differences in dosage of clarithromycin and isotretinoin.
Barbalho, 2021 [31]	Systematic review	- Curcuma and its derivatives were shown to have anti-inflammatory and antioxidant effects. - Curcuma was indicated to increase collagen, facial elasticity, and decrease skin fungus. - Curcuma was also found to reduce erythema, scaling/duration of lesions, and axillary hair growth with possible anti-androgenic effects.
Dall’Oglio, 2021 [32]	Open-label trial	- Treatment with 15% topical azelaic acid (AzA) combined with 1% dihydroavenanthramide D for 8 weeks resulted in significantly improved symptoms in rosacea patients. - Treatment group had decreased Investigator Global Assessment scores (*p* < 0.01); decreased inflammatory lesions; and reduced erythema-directed digital photography scores.
Al-Oudah, 2022 [33]	Clinical trial	- Treatment with 100 mg oral CoQ10 adjuvant in conjunction with the biological medication Adalimumab resulted in a significant improvement of psoriasis, as measured by PASO and DLQI scores (*p* < 0.01). Indicates the antioxidant therapy may have benefits for psoriatic patients when given for 3 months.
Xu, 2022 [34]	Controlled trial	- In IMQ-induced psoriatic mice, the group treated with topical astilbin had less skin scaling, thickening, and erythema compared with the calcipotriol-treated and control groups. Additionally, astilbin significantly reduced pathological changes when examined histologically. - Administration of calcipotriol or astilbin resulted in significantly more mild psoriatic symptoms compared with the group receiving IMQ only. - Calcipotriol and astilbin both increased IMQ-induced keratinocyte differentiation and proliferation (which is increased in the psoriatic model). - Activation of BMDCs and inflammation factors such as IL-17 were significantly reduced in models treated with astilbin.
Bahraini, 2018 [35]	Randomized controlled trial	- In a randomized, placebo-controlled trial, groups receiving the oral turmeric tonic displayed significantly higher improvements in quality of life amongst psoriatic patients. - The turmeric tonic demonstrated significant decrease in PASI scores as well as in erythema, scaling, and thickness of skin compared with the placebo group.
Mason, 2013 [36]	Systematic review	- Topical vitamin D analogues were shown to be effective for psoriatic patients compared with placebo group, given as becocalcidiol and paricalcitol. - Corticosteroids, Dithranol, vitamin D with corticosteroids, and tazarotene were all shown to outperform the placebo group. - For psoriasis on the body and scalp, combined topical vitamin D and corticosteroid treatment improved symptoms more than either agent alone. - Psoriasis on the scalp symptoms improved more when group was given potent to very potent corticosteroids compared with vitamin D, and also resulted in fewer adverse effects.
Al-Katib, 2018 [37]	Randomized controlled trial	- Oral vitamin C supplementation directly correlated with GSH levels - Oral vitamin C has demonstrated insignificant effects against psoriasis
Ahmad, 2023 [38]	Randomized controlled trial	- In this randomized clinical trial, groups were given Majoon Ushba (MU) with Marham Raal (MR) ointment or topical terbinafine hydrochloride. Both groups resulted in a significant reduction in symptoms of tinea corporis, including erythema, scaling, and itching—however, margin- and lesion-size changes were insignificant. - Statistically significant difference in mycological cure between both groups and the baseline, but with no significant difference between both test groups.
Iraji, 2022 [39]	Randomized controlled trial	- In patients with mild–moderate eczema, both groups receiving mometasone 0.1% or an herbal cream demonstrated significant reductions in SCORAD value. No significant difference between both test groups.
Javanbakht, 2010 [40]	Randomized controlled trial	- Administration of vitamin E for 60 days resulted in significantly increased alpha-tocopherol levels. - No significant difference found in SCORAD between groups receiving vitamin D only and placebo. However, compared with the baseline group, groups receiving vitamin D, E, and D and E demonstrated significant reductions in SCORAD (34.8%, 35.7%, and 64.3%, respectively). - The group receiving vitamins D and E showed the greatest change in symptoms (62%, *p* = 0.001).
Maralit Bruan, 2019 [41]	Clinical trial	- Treatment with 4% Hibiscus rosa-sinensis leaf extract ointment for <12 wks. resulted in complete ulcer closure in 10 patients (83.3%)
Panahi, 2012 [42]	Randomized controlled trial	- Curcumin supplementation resulted in decreased substance P activity and increased SOD, glutathione peroxidase, and catalase activity compared with the placebo group. - Supplementation resulted in decreased SCORAD and VAS values with less pruritus.
Woolery-Lloyd, 2010 [43]	Randomized controlled trial	- Topical Sodium L-ascorbyl-2-phosphate (APS), a vitamin C derivative, administration demonstrated significantly decreased inflammatory and non-inflammatory lesions. Statistically significant results appeared at week 8. - The group receiving the APS agent had statistically significant reductions in Global Assessment Score by the 8th week.
Shubber, 2020 [44]	Randomized controlled trial	- Combination of doxycycline (100 mg/d) plus vitamin C (500 mg/d) was more efficient as therapeutically in comparison to doxycycline alone (100 mg/d)
Klock, 2005 [45]	Clinical study	- 1% sodium ascorbyl phosphate (SAP) reports a log reduction of 5 after 8h on P. acnes in a time-kill study - SAP reduces lipid oxidation in vivo
Kus, 2005 [46]	Clinical trial	- Vit E does not reduce incidence nor severity of side-effects related to isotretinoin in treatment of acne vulgaris

**Table 3:** Summary of studies retrieved to remark on the anti-skin-cancer utility of antioxidant supplementation (n = 14).

Author, Year	Study Design	Key Findings
Godic, 2014 [47]	Review	- UV radiation causes an increase in the level of reactive oxygen species (ROS) within an individual, which can lead to oxidative stress if the ROS overcome antioxidant barrier effects. Oxidative stress is linked to cancer - Studies have shown that changes in diet by incorporating more antioxidant-containing nutrients may help to reduce oxidative stress and prevent skin cancer. Antioxidants like vitamins C and E are not able to be produced by the human body, so are especially relevant in considering a diet to reduce oxidative stress
Poswig, 1999 [48]	Randomized controlled trial	- Upon prolonged exposure to UV radiation, adaptative protective measures have been observed, specifically a significant increase in manganese-superoxide dismutase (MnSOD) on both mRNA and protein levels. MnSOD works to reduce the superoxide anion (O_2_), thus lowering ROS levels and detoxifying the cell post-UV exposure. Repeated UV exposure was found to increase the MnSOD response, further supporting the idea of MnSOD as an adaptive antioxidant protection response
Fuchs, 1989 [49]	Randomized controlled trial	- Post-UV radiation on mice (associated with an increase in ROS levels), the most significant diminishing effect was observed in catalase activity in skin, indicating cutaneous catalase may be more susceptible to the effects of UV exposure than other antioxidants. Inhibition of catalase has been shown to enhance the damaging effect of oxidative stress
Elmore, 2005 [50]	Review	- Ascorbic acid, also known as vitamin C, normally functions as an antioxidant and is generally not damaging to DNA. However, when combined with certain metal ions (such as with sodium in Sodium Ascorbate), ascorbic acid may exhibit a pro-oxidant effect that is DNA-damaging - Given that ascorbic acid is a common cosmetic ingredient, manufacturers should be aware of the effect of mixing it with metals
Katiyar, 2005 [51]	Randomized controlled trial	- Due to the toxicity accompanying the incomplete solar protection sunscreens offer, the need for a more effective and practical method of protection from UV radiation could be satisfied through consumption of the naturally occurring antioxidant silymarin - Current experiments suggest that topical application of silymarin has been effective in preventing melanoma induced by UVB (a high energy component of UV radiation) in animal models, and that consumption of silymarin has protected against the effects of chemical carcinogenesis in animal tumor models. Next steps of testing silymarin in human models are promising
Cho, 2010 [52]	Randomized controlled trial	- Beta-carotene has been shown to have dual antioxidant and pro-oxidant effects, making its overall use as a potential cancer-protective mechanism an area for investigation - In women, only in low-doses did beta-carotene have a helpful effect, increasing type I procollagen mRNA levels, reducing the appearance of wrinkles, and reducing thymine dimer staining, which is associated with UV-induced toxicity
Nishino, 2004 [53]	Randomized controlled trial	- Carotenoids and flavonoids are dietary antioxidants shown to be preventative against cancer. Furthermore, mice with hepatoma (liver cancer) treated with a carotenoid mix exhibited suppressed cancer development - Mice given tumor-promoting nitric oxide (NO) exhibited suppressed tumor development when treated with curcumin and nobiletin
Katiyar, 2003 [54]	Randomized controlled trial	- Green tea contains polyphenols which are antioxidative in nature. These polyphenols (GTP) have been shown to protect against both chemically induced and UV-induced cancers by reducing oxidative stress and inflammation indicative of carcinogenesis - Of the GTP, (-)-epigallocatechin-3-gallate (EGCG) seems to be the most effective as protection against cancer
van der Pols, 2009 [55]	Prospective cohort study	- Not all antioxidants act equally in cancer prevention. From the antioxidants of carotenoids, alpha-tocopherol, and selenium, only selenium supplementation via topical serum was found to be associated with lower tumor incidences of basal or squamous cell carcinoma
Le Gal, 2015 [56]	Randomized controlled trial	- NAC and vitamin E analog Trolox increased migration and invasiveness of human malignant melanoma cells - NAC and vitamin E analog Trolox increased activation of small guanosine triphosphatase RHOA
Hercberg, 2007 [57]	Randomized controlled trial	- The effect of antioxidant supplementation on the incidence of skin cancer (SC) varies with gender, with women displaying a higher percentage of associated SC and melanomas upon antioxidant supplementation than men - The gendered response to antioxidants may have to do with gender differences in nutrient metabolism and/or gender differences in pre-supplementation antioxidant presence in the skin due to diet
Kashif, 2023 [58]	Randomized controlled trial	- VitC, β-carotene, retinyl palmitate, and canthaxanthin (all found in food) promote the activation of transcription factor BACH1 in mice, which seems to be associated with mouse melanoma metastasis, particularly in the liver and lymph node - Therapeutic treatment of metastasis seems likely to involve pro-oxidant rather than antioxidant strategies
Piskounova, 2015 [59]	Randomized controlled trial	- Studies show contradictory effects of antioxidants on carcinogenesis; however, here, Piskounova finds that dietary supplementation of antioxidants actually promotes metastasis (the spread of cancer), particularly breast cancer, in the case of the antioxidant folate - Conversely, oxidative stress was found to limit the incidence of melanoma metastasis and tumor formation
Ezzedine, 2010 [60]	Randomized controlled trial	- In a 5-year follow-up after supplementing individuals with a daily antioxidant cocktail (containing ascorbic acid, vitamin E, beta-carotene, selenium, and zinc) for nine years, a lower risk of skin cancer was observed in the follow-up as compared with during the intervention, suggesting a potential cancer-promoting role for antioxidants

**Table 4:** Summary of studies retrieved to remark on emerging evidence for antioxidant supplementation for dermatologic conditions (n = 9).

Author, Year	Study Design	Key Findings
Burke, 2022 [61]	Randomized controlled trial	- In the ascorbic acid group, 86.7% of lesions showed complete resolution after 8 weeks, while in the IMQ group, only 57.1% of lesions were resolved (*p* < 0.05 Chi Square), indicating that topical ascorbic acid was superior to topical imiquimod in treating low-risk nodular and superficial lesions at 8 weeks and non-inferior at 12 weeks.
Huang, 2020 [62]	Meta-analysis	- Imiquimod has a clinical and histological clearance rate of over 70% for nBCC, with a low recurrence rate of 1.80%, making it a viable treatment option for patients who decline or are not suitable for surgical intervention, despite its lower clearance rates compared with surgery.
Bánvölgyi, 2020 [63]	Controlled trial	- In a small group of patients with extensive BCCs, IVA was found to be well-tolerated, but with the availability of smoothened receptor inhibitors, it may only be used as an adjuvant therapy for treatment-resistant cases.
Neville, 2007 [64]	Controlled trial	- Post-treatment excision showed no histologic evidence of residual tumor in all 17 lesions (100%). Although most patients (67%) experienced local site reactions that required a rest period from medication application, the majority of them preferred this treatment over excision if they developed a subsequent tumor.
Shumack, 2002 [65]	Randomized controlled trial	- In both the 6- and 12-week studies, dosing topical 5% imiquimod cream once daily for 7 days per week resulted in the highest clearance rate, with 71% and 76% of patients showing clearance of their tumor, respectively. Thus, it can be concluded that this treatment modality is well-tolerated and highly effective in treating nodular BCC when applied once daily for 7 days per week for either 12 or 6 weeks.
Holló, 2016 [66]	Controlled trial	- Out of the 7 treated lesions, 4 tumors resolved, but tumor recurrence was observed in 1 histologically tumor-free patient during an 18-month follow-up period.
Farajzadeh, 2022 [67]	Randomized controlled trial	- The combination therapy group showed a significant improvement of more than 75% compared with the monotherapy group (0%) at the end of the treatment (*p* = 0.001).
Moludi, 2021 [68]	Randomized controlled trial	- The probiotic group demonstrated significant improvement in total BDI scores (−6.15 ± 2.10 vs. 1.39 ± 1.80, *p* = 0.017) and DLQI (−9.50 ± 4.1 vs. 0.12 ± 0.6, *p* = 0.045), as well as a considerable reduction in PASI and PSS scores (−5.26 ± 3.75 vs. 0.48 ± 1.37, *p* = 0.049 and −4.85 ± 3.10 vs. 0.43 ± 0.80, *p* = 0.047, respectively) compared with the placebo group. The intervention group also showed significant improvements in TAC levels and reductions in hs-CRP levels, IL-6 levels, and MDA levels compared with the placebo group.
Michelotti, 2021 [69]	Randomized controlled trial	- The probiotic mixture improved skin smoothness, moisturization, self-perception, and reduced the SCORAD index and inflammatory markers associated with AD. The selected probiotic strains showed sustained improvement in AD-related symptoms and skin conditions up to T84d.
